# Choosing the source of healthy controls for studies on myeloid malignancies: all bone marrow cells are created equal, but some are more equal than others

**DOI:** 10.1186/s13287-023-03257-z

**Published:** 2023-03-08

**Authors:** Jennifer Rivière, Jennifer Hock, Michèle C. Buck, Judith S. Hecker, Katharina S. Götze, Mark van der Garde

**Affiliations:** 1 Department of Medicine III, School of Medicine, Technical University of Munich, Ismaninger Strasse 22, 81675 Munich, Germany; 2 German Cancer Consortium (DKTK), Partner Site Munich, Ismaninger Strasse 22, 81675 Munich, Germany; 3 German Cancer Research Center (DKFZ), Im Neuenheimer Feld 280, 69120 Heidelberg, Germany

**Keywords:** Hematopoietic stem cells, Mesenchymal stromal cells, Iliac crest, Femoral head, Healthy control

## Abstract

Bone marrow samples from discarded femoral heads are often used as healthy controls in studies investigating the in vitro characteristics of cells from patients with hematologic malignancies. Since patient samples are usually derived from iliac crest aspirates, this carries the risk that the properties of the cells from both sources might be different due to the site and method of harvesting. Comparing BM cells from iliac crest aspirates and femoral heads from age-matched healthy donors, we show that, while mesenchymal stromal cells have indistinguishable properties between both sources, hematopoietic stem and progenitor cells (HSPC) from femoral heads show a considerable proliferative advantage in vitro. These data therefore suggest that experiments comparing leukemic cells from the iliac crest to healthy HSPC obtained from femoral heads should be interpreted with caution.

**Supplementary Information:**

The online version contains supplementary material available at 10.1186/s13287-023-03257-z.

## Background

Femoral heads (FHs) from hip replacement surgery provide a rich source of healthy bone marrow (BM) that is often used as a control sample in experimental set-ups for BM obtained from patients with hematologic malignancies [1–5]. Although phenotypically identical, patient BM is usually obtained from iliac crest aspirates (ICAs). The assumption that the properties of ICA BM cells are similar to those from FHs carries the risk that data may be misinterpreted, in particular if the observed differences should actually be attributed to the site and method of BM harvest.

In young adult mice the site of BM does not influence the properties of hematopoietic stem and progenitor cells (HSPC) [6]. However, with ageing, the majority of hematopoiesis shifts from the long bones to the pelvis, sternum, skull and vertebrae [7] with a concomitant increase in BM adiposity of the femur [8]. This change in the composition of the BM microenvironment has a direct influence on the residing hematopoietic and mesenchymal cells [9], which could particularly be of concern for studies on acute myeloid leukemia (AML) and myelodysplastic syndromes (MDS) since both hip replacement surgery patients, AML and MDS patients are of advanced age and cells are obtained from either the femoral head or the pelvis, respectively. Furthermore, FH BM is kept in the bone prior to processing whereas BM aspirates from the iliac crest are stored in a syringe, tube or bag, which can affect the quality and properties of the cells [10].

## Materials and methods

The materials and methods used in this study can be found in the additional file online.

## Results

In order to shed light on this issue, we performed functional analyses of BM samples from healthy donors < 60 years, obtained by either aspiration from the iliac crest or from femoral heads following hip replacement surgery. Using age-matched samples (Additional file 1: Fig. S1), we studied multiple in vitro characteristics of both HSPC and mesenchymal stromal cells (MSC) and indeed found significant differences in the properties of these cells. These findings could have important consequences for the interpretation of existing data and design of future studies using FH as BM controls.

**Fig. 1:**
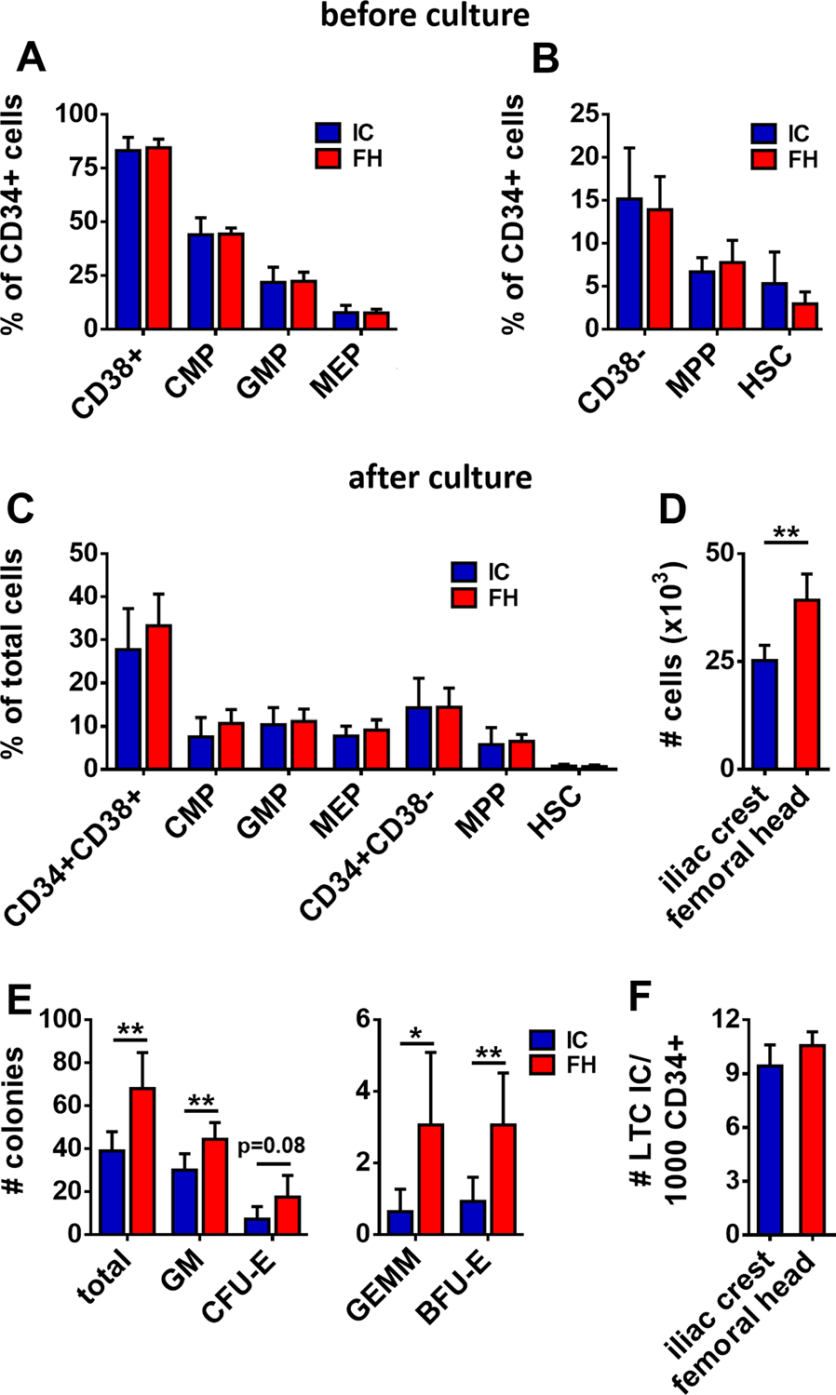
Analysis of the proportion of HSPC populations of CD34^+^ cells from the ilium or femur. **A**, **B** Proportion of each population, before culture, as a percentage (mean ± SD) of the CD34^+^ cells. CMP: common myeloid progenitor, GMP: granulocyte monocyte progenitor, MEP: megakaryocyte erythrocyte progenitor, MPP: multipotent progenitor, HSC: hematopoietic stem cell. **C** Proportion of early progenitors and myeloid progenitors after 7 days of culture in stem cell differentiation medium (mean percentage ± SD). **D** Number of cells (mean ± SD) after 7 days of culture of 1 × 10^4^ CD34^+^ cells. **E** Number (mean ± SD) of different myeloid colonies after 14 days of culture in complete methylcellulose medium. *GM* granulocyte/monocyte,* CFU-E* colony forming unit erythrocyte,* GEMM* granulocyte/erythrocyte/monocyte/megakaryocyte,* BFU-E* burst forming unit erythrocyte. **F** Number of colonies (mean ± SD) obtained after Long-Term Culture Initiating Cell (LTC IC) assay per 1000 CD34^+^ cells

We first analyzed the proportion of early hematopoietic progenitors in CD34^+^ cells isolated from ICA or FH BM by flow cytometry (Additional file 1: Fig. S2). Small differences in HSPC proportions could be found between different donors, but on average there were no significant differences between both sources (Fig. 1A, B), suggesting that neither the site of harvest nor the method of extraction of the BM affects the composition of the BM HSPC pool.

We next assessed the proliferation and differentiation capacity of HSPC, by performing proliferation cultures and colony-forming-unit (CFU) assays. After 7 days of suspension culture in serum-free medium supplemented with cytokines, we again could not detect any differences in the proportions of different progenitor populations between the cells from ICAs or FHs (Fig. 1C). However, expansion of HSPC from FHs was significantly higher compared to ICA HSPC, as shown by the total cell number after 7 days (Fig. 1D, 3.9 ± 0.6-fold expansion vs. 2.5 ± 0.4-fold expansion, respectively, *p* = 0.008). A similar observation was made after seeding CD34^+^ cells in methylcellulose. After 14 days of culture, HSPC from the femur formed a significantly higher number of colonies (Fig. 1E, 68.1 ± 16.7 colonies/1000 CD34^+^ cells from FHs vs. 39.0 ± 9.0 colonies for CD34^+^ cells from ICAs, *p* = 0.006). The number of all types of colonies was increased significantly for CD34^+^ HSPC from FHs (Fig. 1E), with the exception of the colony foming unit erythrocyte (CFU-E) due to a high variability between samples. Notably, regardless of clonogenic capacity, the proportion of granulocyte/erythrocyte/monocyte/megakaryocyte (GEMM) colonies from FH CD34^+^ HSPC was almost threefold higher than from ICA CD34^+^ HSPC (Additional file 1: Fig S3, 4.3% ± 2.7% vs. 1.5% ± 1.5%, *p* = 0.02). Since GEMM colonies are known to originate from cells that are higher up in the hematopoietic stem cell hierarchy [11], this could suggest that CD34^+^ cells from FHs contain a higher number of primitive HSPC. However, this was not reflected by the immunophenotype, showing that CD34^+^ cells from ICAs had a slightly higher percentage of HSC than CD34^+^ cells from FHs, albeit not significant (Fig. 1B, 5.3% ± 3.7% vs. 3.0% ± 1.4%, *p* = 0.22). To investigate the stem cell potential of CD34^+^ cells, we performed long-term culture (LTC) assays, a surrogate in vitro assay for stem cell activity. After 6 weeks of culture on a stromal layer, followed by replating in methylcellulose, FH CD34^+^ HSPC generated a slightly higher number of colonies (Fig. 1F, 10.6 ± 0.7 colonies vs. 9.4 ± 1.2 colonies/1000 CD34^+^ cells for FHs and ICAs, respectively, *p* = 0.10), indicating an increase in more primitive HSPC with self-renewal properties. However, considering the limited size of this increase, this difference is not likely to affect comparative studies.

**Fig. 2:**
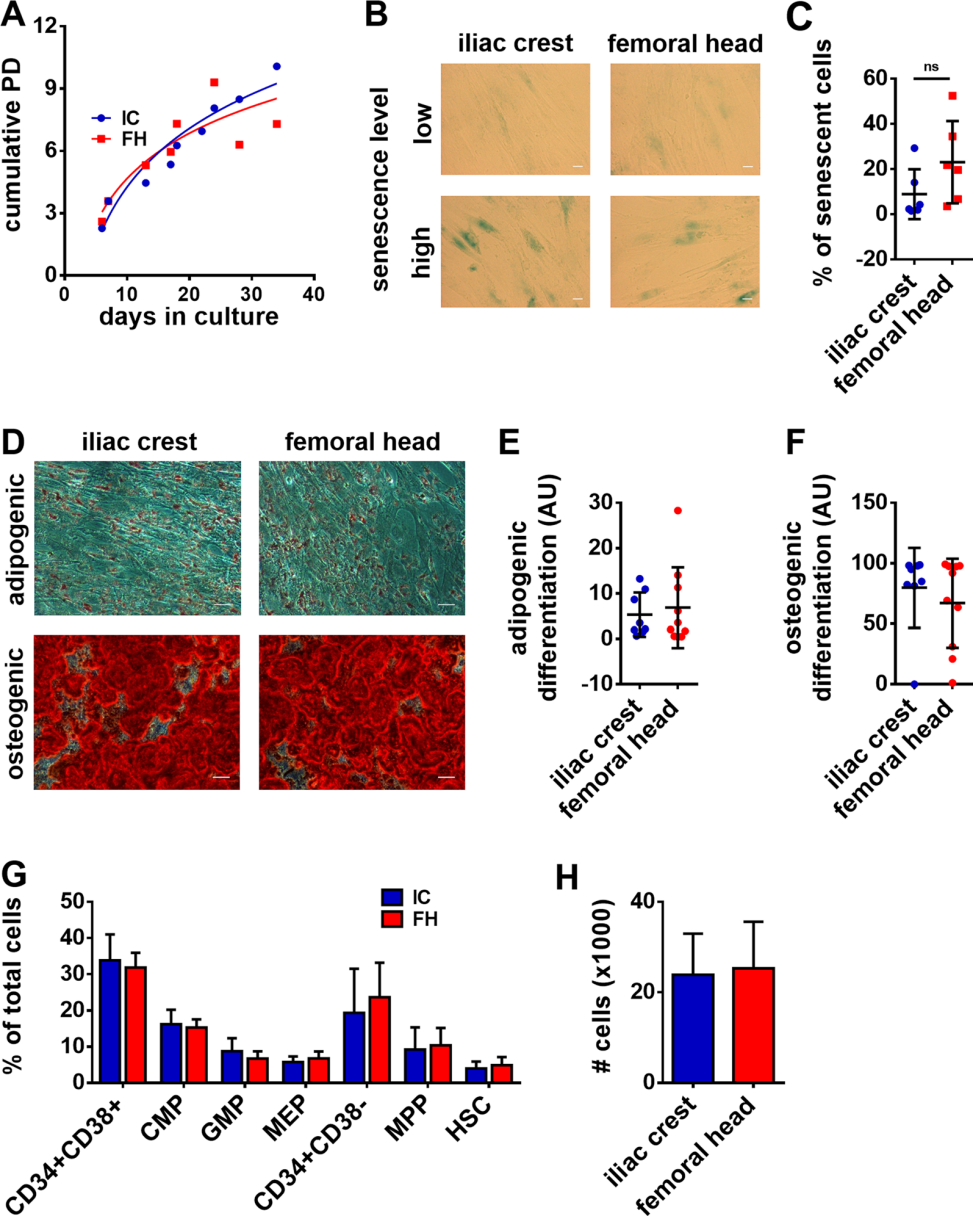
Analysis of the functional properties of MSC cultured from iliac crest aspirations and femoral heads. **A** The cumulative population doubling (PD) of MSC is presented according to the number of days since the beginning of cell culture (mean). **B** Representative pictures of β-galactosidase staining of MSC at passage P6 from samples with low level of senescence (top) or high level of senescence (bottom), scale bar = 20 µm. **C** Quantification of the percentage of senescent cells at passage P6 (mean ± SD, ns = non significant using a Mann–Whitney test). **D** Representative pictures of adipogenic (top) and osteogenic (bottom) differentiation capacity at passage P3, scale bar = 50 µm. **E**, **F** Quantification of adipogenic and osteogenic differentiation capacity (mean ± SD, arbitrary units). **G**, **H** Proportions of HSPC subpopulation (**G**) and number of cells (**H**) after 4 days of co-culture of HSPC and MSC from the iliac crest or femoral head (mean percentage ± SD)

Finally, we compared the stromal compartment from FHs and ICAs BM samples, using MSC isolated from the mononuclear cell fraction by plastic adherence. MSC from both sources were negative for CD45 and CD34, and positive for the characteristic MSC markers CD90, CD105 and CD73 (Additional file 1: Fig. S4). Analysis of the proliferation capacity of MSC cultured from ICAs and FHs showed no significant differences in their cumulative population doubling (Fig. 2A). To determine whether MSC from either BM source had a higher tendency to display features of ageing, we performed senescence assays on cells of passage 6 using the β-galactosidase assay (Fig. 2B, C). A higher mean percentage of senescent cells for FH MSC (23.1% ± 18.2) was observed compared to ICA MSC (8.9% ± 11.0), but due to a high variability between samples from each group, this difference was not significant.

Adipogenic and osteogenic differentiation has been shown to be dysregulated in diseases, such as MDS and AML [12]. We therefore analyzed the differentiation potential of MSC from ICAs and FHs into adipogenic and osteogenic lineages. Image-based quantification of the staining of lipid droplets and calcium deposits showed a similar differentiation potential into both lineages (Fig. 2D-F). Lastly, since MSC provide vital support for HSPC homeostasis in the BM, we examined the capacity of FH and ICA MSC to support HSPC culture. Flow cytometry analysis of HSPC after culture showed no differences in the proportion of HSPC subpopulations (Fig. 2G) or HSPC number (Fig. 2H), indicating that potential to provide the hematopoietic support of MSC from both BM sources is approximately equal.

## Discussion

In summary, while MSC from both ICAs and FHs seem to proliferate, differentiate and support HSPC in a similar manner, BM CD34^+^ cells from FHs expand at a higher rate and contain more CFU, in particular immature CFU, compared to BM CD34^+^ cells from ICAs. These differences may be explained by intrinsic factors (e.g., a higher proliferation rate or a less quiescent state of HSPC) but could also be caused by extrinsic factors (e.g., differences in storage and processing methods including mechanical stress). In the BM, HSPC reside in a hypoxic microenvironment and exposure to oxygen can induce a loss of stem cell potential [13], which is demonstrated by the fact that isolation of HSPC from BM or cord blood under hypoxic conditions increases the number of primitive HSPC [14]. Furthermore, mechanical stress can induce differentiation and a loss of primitive HSPC (15). Indeed, since ICA HSPC are stored in a collection bag, it is likely that they are exposed to higher levels of oxygen and mechanical stress, while FH HSPC remain in their natural microenvironment until mononuclear cell isolation. We hypothesize that this exposure could explain the lower proliferation capacity of ICA HSPC.

## Conclusions

In conclusion, although FHs remain an attractive source of healthy BM due to their widespread availability and relatively low cost, our in vitro results indicate that CD34^+^ cells from FHs are not entirely comparable to patient samples obtained from ICAs as they show higher proliferative capacity of HSPC. Thus, in vitro studies using CD34^+^ HSPC from FHs as healthy controls in experimental set-ups examining myeloid malignancies should be interpreted with caution. Moreover, in future it would be advisable to obtain healthy BM samples from sources that more closely resemble the harvesting and processing protocols of patient BM.

## Supplementary Information

**Additional file 1.** Supplementary figures S1–S4 and supplementary materials and methods.

## Data Availability

Data sharing is not applicable to this article as no datasets were generated or analyzed during the current study. Access to data files and further information is available upon request by contacting Mark van der Garde, mark.garde@tum.de, tel. + 49 89 4140 6318.

## Abbreviations

AML: Acute myeloid leukemia
BM: Bone marrow
CFU: Colony-forming unit
CFU-E: Colony-forming unit erythrocyte
CFU-GEMM: Colony-forming unit granulocyte, erythrocyte, monocyte, megakaryocyte
FH: Femoral head
HSC: Hematopoietic stem cell
HSPC: Hematopoietic stem and progenitor cell
ICA: Iliac crest aspirate
LTC: Long-term cell culture
MDS: Myelodysplastic syndrome
MSC: Mesenchymal stromal cell
