# Supplementary material for: Choosing the source of healthy controls for studies on myeloid malignancies: all bone marrow cells are created equal, but some are more equal than others
Source: Stem Cell Res Ther. 2023 Mar 8;14:36. doi: 10.1186/s13287-023-03257-z (PMC9993599; doi:10.1186/s13287-023-03257-z)
Supplement: Supplementary file 1 — Additional file 1. Supplementary figures S1–S4 and supplementary materials and methods. [file 13287_2023_3257_MOESM1_ESM.pdf]

Supplementary File

Choosing the Source of Healthy Controls for Studies on Myeloid Malignancies: All Bone Marrow Cells are Created Equal, but Some are More Equal than Others.

Jennifer Rivière[1†], Jennifer Hock[1†], Michèle C. Buck[1], Judith S. Hecker[1], Katharina S. Götze[1,2,3], Mark van der Garde[1,2,3]

[1] Technical University of Munich, School of Medicine, Munich, Germany

[2] German Cancer Consortium (DKTK), partner site Munich, Germany.

[3] German Cancer Research Center (DKFZ), Heidelberg, Germany

[†] These authors contributed equally to this work

Supplementary figures:

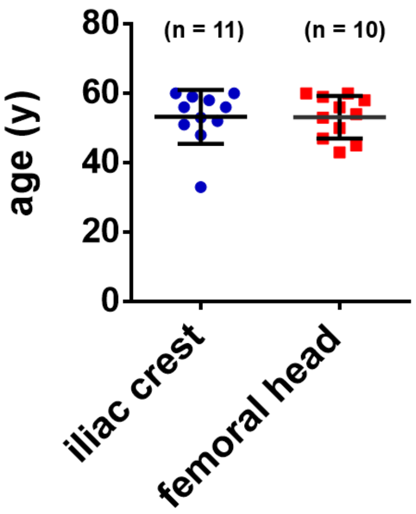

Supplementary figure 1: Donor age of the samples used in this study.

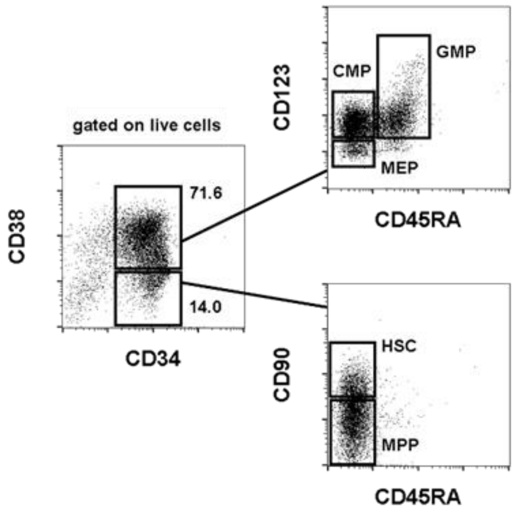

Supplementary figure 2: Gating strategy for the flow cytometry analysis of HSPC subpopulations. CMP: common myeloid progenitor ($CD34^+CD38^+CD123^+CD45RA^-$), MEP: megakaryocyte-erythrocyte progenitor ($CD34^+CD38^+CD123^-CD45RA^-$), GMP: granulocyte-monocyte progenitor ($CD34^+CD38^+CD123^+CD45RA^+$), HSC: hematopoietic stem cell ($CD34^+CD38^-CD90^+CD45RA^-$), MPP: multipotent progenitor ($CD34^+CD38^-CD90^-CD45RA^-$). FMOs (Fluorescence minus one) were used as controls.

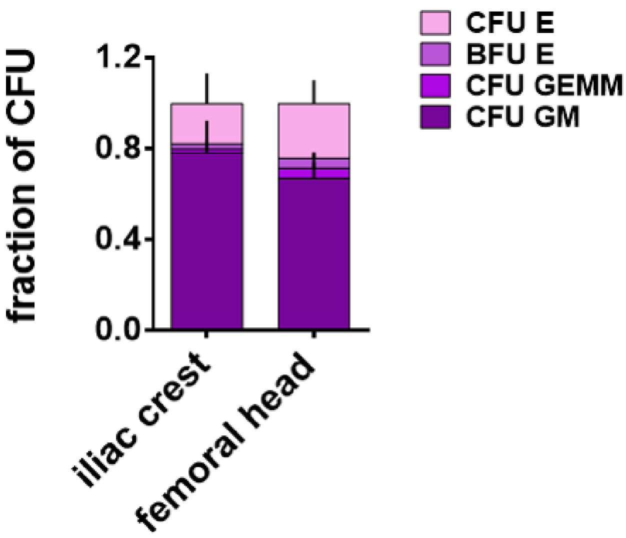

Supplementary figure 3: Fraction of each colony type after CFU culture as a proportion of the total number of colonies. CFU GM: granulocyte, monocyte colonies, CFU GEMM: granulocyte, erythrocyte, monocyte, megakaryocyte, BFU E: burst forming unit erythrocyte, CFU E: colony forming unit erythrocyte.

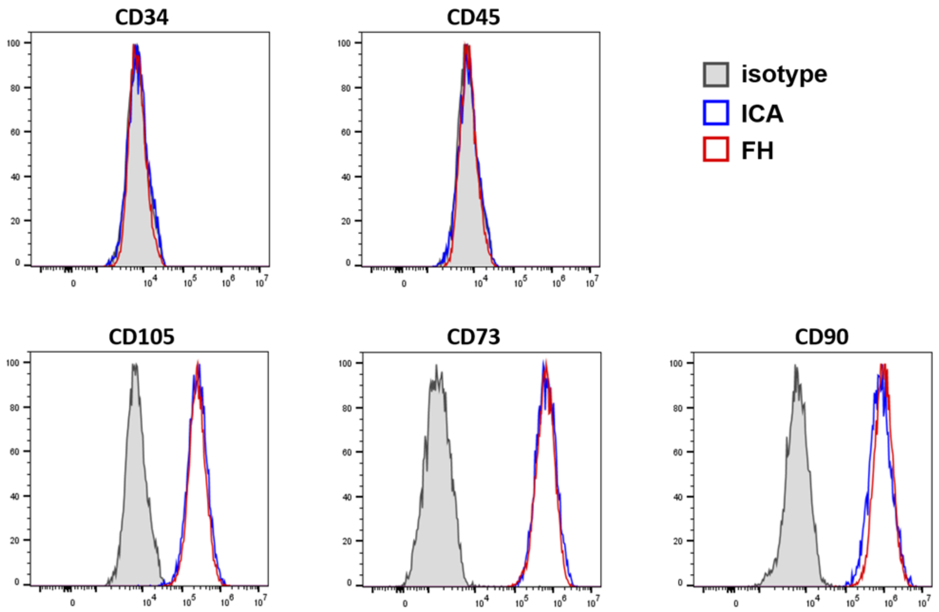

Supplementary figure 4: MSC marker expression. Representative flow cytometry plots of common MSC markers. Blue line: MSC from iliac crest aspirate (ICA), red line: MSC from femoral heads (FH), grey: FMO control.

Supplementary materials and methods

Human bone marrow samples

Femoral heads (FHs) were obtained from healthy donors undergoing hip replacement surgery (Dr. Martin Nolde and Dr. Dominikus Hausmann, OZB Munich). Iliac crest aspirates (ICAs) were obtained from excess BM collection bags of allogeneic donors from the Bayerische Stammzellbank, Gauting. Sample collection was performed with permission of the ethic committee and after informed consent according to the declaration of Helsinki. Donor age distribution is shown in Supplementary Figure 1.

Sample collection, processing, HSPC and MSC isolation

ICAs and FHs were collected at the Bayerische Stammzellbank and the Sana Clinic for Orthopedics and Trauma Surgery, respectively, and transported by courier to the laboratory.

ICAs were stored in a closed system BM collection bag and FHs were stored in sterile polyethylene bags (Whirl-Pak sample bag, #1-7162, Neolab, Heidelberg, Germany) at 4°C until processing. All samples were processed within 18 hours after collection. Cells from FHs were extracted by mincing the trabecular bone and washing the pieces with PBS. Cells from the ICAs were obtained by backward flushing of the collection bags with PBS. Mononuclear cell (MNC) suspensions were prepared by density gradient separation (Biocoll Separating Solution density 1.077g/ml, Bio-sell, #BS.L 6115). Cells were cryopreserved in freezing medium containing 45% Iscove's Modified Dulbecco's Medium (IMDM) with GlutaMAX (Gibco, #31980-048, Thermo Fisher, Germering, Germany), 45% FBS (Gibco, #10270106, Thermo Fisher) and 10% DMSO (#20385, Serva, Heidelberg, Germany) and stored in liquid nitrogen until further use. $CD34^+$ cells were isolated by magnetic bead separation (CD34 Microbead Kit, #130-046-703, Miltenyi Biotec, Bergisch Gladbach, Germany). MSC were isolated by plastic adhesion of MNC plated in normal MSC medium containing α-MEM (#M4526, Sigma-Aldrich), 20 U/ml penicillin–streptomycin, 2 mM L-glutamine, 10 U/L heparin (#H3149, Sigma-Aldrich), and freshly added 10% (v/v) pooled human platelet lysate (described in [1]). Half-medium changes were realized twice a week and MSC were passaged when confluency was reached. MSC experiments were started after passage 2.

Flow cytometry analysis of hematopoietic stem and progenitor cells (HSPC)

At day 0, $CD34^+$ enriched MNC cells from FHs and ICAs were stained with the following antibodies: anti-CD38 e450 (clone HIT2, Invitrogen, #48-0389-42, Thermo Fisher), anti-CD123 APC (clone 6H6, Invitrogen, #17-1239-42, Thermo Fisher), anti-CD45 APC-Cy7 (clone HI30, Biolegend, #304014, San Diego, USA), anti-CD34 FITC (BD Biosciences, #555821, Heidelberg, Germany), anti-CD45RA PE-Cy7 (clone HI100, BD biosciences, #560675), anti-CD90 PE (BD biosciences, #555596). Dead cell exclusion was realized using 1µg/ml DAPI (Biolegend, #422801). Absolute cell count was performed using Flow-count Fluorospheres (Beckman Coulter, #7547053). Cells were analyzed on a CyAn ADP flow cytometer (BeckmanCoulter), equipped with 405nm, 488nm, and 633 nm lasers. FlowJo v10 was used

for data analysis and fluorescence minus one (FMOs) were used as controls to set the threshold of positivity.

HSPC expansion cultures

10,000 CD34$^+$ cells were seeded in triplicate in 96-well plates with serum-free medium composed of IMDM with GlutaMAX (Gibco, #31980-22) and 20% of serum substitute BIT 9500 (Stem Cell Technologies, #09500), supplemented with 100 ng/ml SCF (PreproTech, #300-07), 100 ng/ml Flt3L (PeproTech, #300-19), 25 ng/ml TPO (PreproTech, #300-18), 10 ng/ml IL-6 (PreproTech, #200-06) and 10 ng/ml IL-3 (PreproTech, #200-03). Cells were cultured for 7 days at 37°C and 5% CO_2. All wells were harvested and analyzed for cell numbers and the proportion of hematopoietic progenitors with flow cytometry (as described above).

Colony Forming Unit (CFU) assays

1,000 CD34$^+$ cells were seeded in 1ml of complete methylcellulose medium (StemMACS HSC-CFU with EPO, Miltenyi Biotec, # 130-091-280) in duplicate and cultured at 37°C and 5% CO_2. After 14 days all colonies were counted and classified by standard morphologic criteria.

Long Term Culture (LTC) assays

10,000 CD34+ cells were seeded on a one week-old monolayer of 30Gy irradiated EL08 stromal cells [2] and cultured in Myelocult H5100 medium (Stem Cell Technologies, #05150) containing 10 ng/ml Flt3L, 20 ng/ml TPO and 1µM hydrocortisone for 6 weeks after which all cells were transferred in complete methylcellulose medium (StemMACS HSC-CFU with EPO, Miltenyi Biotec, # 130-091-280). Colonies were counted after 14 days. .

MSC expansion assay

MSC were seeded at 5000 cells/cm² and cultured until confluency. MSC expansion was assessed by counting total cell number harvested after trypsinization, and subsequently plated for the next passage expansion analysis. Population doubling was calculated using the

following formula: PD=log2(Nc/Ni) where Nc is the number of cells harvested at confluence, and Ni is the initial number of plated cells.

MSC senescence assay

40,000 MSC from passage 6 were seeded in 24-well plates in normal MSC medium and cultured for 4 days. β-galactosidase enzymatic activity at pH 6 was used to assess senescence using the Senescence cells histochemical staining kit (#CS0030, Sigma-Aldrich) following manufacturer's instructions. After staining, pictures were acquired with a light microscope (Axiovert 25, Carl Zeiss Microscopy, Jena, Germany) with a 20X objective (Olympus Germany, Model: CKX41, 20X objective model: LCAch N 20X.0.40 PhP ∞/1/FN22, no filters were used for the acquisition) and analyzed using ImageJ software (version 1.52a) for quantification of total cells and cells developing a blue coloration indicating senescence onset.

MSC differentiation assay

60,000 MSC were seeded in 24-well plates in normal MSC medium. On the following day, medium was replaced for either adipogenic-inducing differentiation medium (normal MSC medium supplemented with 1µM Dexamethasone (#D4902 Sigma-Aldrich), 60µM Idomethacine (#I7378 Sigma-Aldrich), 500µM IBMX (#I5879 Sigma-Aldrich), 10µM Insuline (#91077C, Sigma-Aldrich)) or osteogenic-inducing differentiation medium (#PT-3002, Lonza). MSC were cultured for 18 days in the respective differentiation mediums with half-medium changes performed twice a week. Subsequently, differentiated MSC were stained with Oil-Red-O (#09755, Sigma-Aldrich) solution or Alizarin Red S solution (#A5533, Sigma-Aldrich) for adipogenic and osteogenic staining, respectively, as previously described [2]. Stained samples were analyzed with a light microscope with a 10X objective (Olympus Germany, Model: CKX41, 10X objective model: CAch N 10X / 0.25 PhP ∞/ - /FN22, Phase contrast slider: IX2-SLP, no filters were used for the acquisition) and at least four random microscopic fields were acquired with a 1.4 megapixel Axiocam ICc1 camera and digitized using AxioVision Software (Carl Zeiss Microscopy, Jena, Germany). No resolution enhancement or averaging

was done during processing of the images. For quantification of staining intensity, the differentiated areas were identified by applying a color threshold, which allowed the identification of red pixels only. The percentage of red pixels (percentage of differentiated area) was normalized to a baseline calculated from undifferentiated stained control samples.

HSPC support by MSC

60,000 MSC were seeded in 24-well plates in normal MSC medium. The following day medium was exchanged for HSPC expansion medium (as described above in HSPC expansion section with the exception that IL3 and IL6 are omitted). After 4 days of co-culture, hematopoietic cells were harvested and analyzed for cell number and proportion of HSPC by flow cytometry

Statistical analysis

P-values were calculated using GraphPad Prism using a Mann-Whitney test unless stated otherwise.

Supplementary references

1. Schallmoser, K. and D. Strunk, *Preparation of pooled human platelet lysate (pHPL) as an efficient supplement for animal serum-free human stem cell cultures.* Journal of visualized experiments: JoVE, 2009(32).

2. Oostendorp, R.A.J., et al. Stromal cell lines from mouse aorta-gonads-mesonephros subregions are potent supporters of hematopoietic stem cell activity. Blood 2002. **99**(4): p. 1183-1189.

3. Weickert, M.-T., et al., *Bone marrow stromal cells from MDS and AML patients show increased adipogenic potential with reduced Delta-like-1 expression.* Scientific reports, 2021. **11**(1): p. 1-12.
